# Utility of [^99m^Tc]Tc-tilmanocept, an immunosuppressive macrophage functional imaging agent in melanoma patients receiving checkpoint inhibitor treatment: a feasibility study

**DOI:** 10.1007/s00262-025-04127-8

**Published:** 2025-09-06

**Authors:** Sarah Boughdad, Sofiya Latifyan, Margret Schottelius, Michael Blue, Stéphanie Tissot, Christine Geldhof, Julien Costes, John O. Prior, Niklaus Schaefer

**Affiliations:** 1 Department of Nuclear Medicine and Molecular Imaging, Lausanne University Hospital, CHUV/UNIL, 1011 Lausanne, Switzerland; 2 Sorbonne Université Santé, 91-105, Bd de L’Hôpital, Paris, France; 3 Department of Oncology, Lausanne University Hospital, Lausanne, Switzerland; 4 Translational Radiopharmaceutical Sciences, CHUV/UNIL, 1011 Lausanne, Switzerland; 5 Agora, Pole de Recherche Sur Le Cancer, 1011 Lausanne, Switzerland; 6 NAVIDEA Biopharmaceuticals, Columbus, OH USA; 7 Department of Oncology, Center of Experimental Therapeutics, Lausanne University Hospital, Lausanne, Switzerland

**Keywords:** Immune checkpoint inhibitors, [^99m^Tc]Tc-Tilmanocept, CD206 imaging, Prediction of response, Quantitative SPECT/CT

## Abstract

**Background:**

Immunotherapy is a mainstay in the treatment of patients with advanced melanoma. Yet, resistance mechanisms exist, and tumour-associated macrophages (TAMs), particularly the M2-like phenotype, are associated with poorer outcomes, with CD206 serving as their specific marker. We present the first human SPECT/CT study to visualize CD206 + TAMs in patients undergoing immunotherapy and compare the findings to clinical outcomes (NCT04663126).

**Material and methods:**

This prospective diagnostic open-label, non-randomized, feasibility study aimed to visualize CD206 + cells including M2-like TAM in target lesions (T-Lesion) of melanoma patients treated with immunotherapy using [^99m^Tc]Tc-Tilmanocept imaging. Patients had dynamic, whole-body planar and SPECT/CT acquisitions at 1- and 3-h after injection of 350 MBq ± 10% [^99m^Tc]Tc-Tilmanocept. SUV_max/peak/mean_, MTV and TLA were measured on SPECT/CT imaging in T-Lesion with ratios to healthy tissues to compare with baseline [^18^F]FDG PET/CT imaging, multispectral immunofluorescence staining findings on lesions’ biopsies, tumour response at three months and follow-up.

**Results:**

Five patients were recruited. T-Lesion uptake on [^99m^Tc]Tc-Tilmanocept imaging remained stable at 1- and 3-h post-injection with strong and significant correlations with baseline [^18^F]FDG PET/CT. SUV_max_ T-Lesion/ SUV_mean_ fat-tissue ratio on [^99m^Tc]Tc-Tilmanocept SPECT/CT at 1-h was significantly associated with tumour response at three months (p = 0.005), total cells densities for macrophages and CD8 + cells on multispectral immunofluorescence staining and poorer outcomes during the follow-up (p = 0.026).

**Conclusion:**

These preliminary pilot data provide the first-in-human proof of concept that CD206-based functional imaging showed measurable signal in tumour lesions in patients with advanced melanoma. If validated it might be useful in reflecting tumour immune status, hence help predicting tumour response to ICI.

**Supplementary Information:**

The online version contains supplementary material available at 10.1007/s00262-025-04127-8.

## Introduction

Modern immunotherapy changed the therapeutic landscape of malignant diseases, especially in patients with advanced melanoma. Particularly notable is the emergence of sustained responses to immune checkpoint inhibitor (ICI) treatment. Yet, many patients do not or insufficiently respond to this treatment, and several attempts to predict outcomes to programmed death-1 receptor (PD1) directed treatment were investigated. Scientific studies reported mutational load (1), neoantigens (2) or PD1/PD1 ligand (PD-L1) expression and/or CD8 lymphocytes infiltrates in melanoma to be predictive for response (3, 4). Recently, non-invasive imaging methods to monitor the expression and distribution of PD1, PD-L1 or the infiltration of cytotoxic T cells expressing CD8 were investigated to select patients eligible for ICI treatment (5, 6).

Similarly, tumour-associated macrophages (TAMs) are important in the immune modulation of the tumour micro-environment (TME) playing an essential role in cancer progression (7, 8). The alternatively activated M2-polarized phenotype is an indicator of poor patients’ outcome. During melanoma progression, the anti-tumoural M1-polarized phenotype might shift towards the M2 phenotype (9), which can suppress immunity (10), leading to the reduction of specific CD8-derived immune response (11). The CD206 receptor or macrophage mannose receptor is a type of C-type lectin expressed mainly by macrophages, dendritic cells and some endothelial cells, and binds to mannose and other sugar residues on the surface of various pathogens (12). CD206-positive (CD206 +) macrophages promote tumour growth, angiogenesis, invasion and metastasis by suppressing anti-tumour immunity and enhancing tumour cell survival (12) through the production of IL10 and TGFbeta, two cytokines that inhibit the activation and function of T cells, NK cells and other immune cells (13).

Tilmanocept is composed of a dextran backbone, modified with multiple mannose moieties to target the CD206 receptor, and includes a DTPA chelator for binding to a radioisotope [^99m^Tc] for single-photon emission tomographysingle-computed tomography (SPECT/CT) imaging. It is currently approved for mapping and localization of tumour draining lymph nodes in breast cancer or melanoma showing superiority to conventional sentinel node procedures (14, 15). Recent study suggested a role of [^99m^Tc]-Tilmanocept in the non-invasive detection of systemic inflammation (16, 17). Due to the specific properties of [^99m^Tc]-Tilmanocept, we hypothesized a possible utility of CD206-directed imaging in patients scheduled or undergoing ICI treatment. This prospective feasibility study investigated [^99m^Tc]Tc-Tilmanocept SPECT/CT imaging in patients prior to or progressing under immunotherapy in comparison with [^18^F]-fluorodeoxyglucose positron emission tomography/computed tomography ([^18^F]-FDG PET/CT) imaging and biopsies findings. We also investigated a potential association between lesion uptake on [^99m^Tc]Tc-Tilmanocept SPECT/CT imaging with tumour response at three months and the outcomes during the follow-up (progressive disease versus no event).

## Material and methods

### Population

In this prospective, single-centre, open-label feasibility study, we included advanced melanoma patients at baseline before first-line ICI treatment or with progressive disease (PD) under ICI defined as clinical progression or progressive disease on clinically indicated imaging. Inclusion criteria were as follows: patient over 18 years old with a biopsy proven melanoma, at least stage III locally advanced or metastatic disease (8th edition AJCC, 18), scheduled to start anti-PD1 treatment after [^99m^Tc]Tc-Tilmanocept imaging, clinically indicated [^18^F]FDG PET/CT scan done within 4 weeks prior to [^99m^Tc]Tc-Tilmanocept imaging. Clinically indicated biopsies were explored for further analysis when available. Exclusion criteria were as follows: pregnant or breastfeeding patients, patients with ocular melanoma, clinical history that could interfere with the study objectives at the discretion of the investigator judgement and known allergy to dextran.

### Imaging protocol

#### [^18^F]FDG PET/CT scan

Patients received 3.5 MBq/kg of [^18^F]FDG after a 6-h fasting period if capillary glycaemia was below 11 mmol/l. [^18^F]FDG PET/CT acquisition with classic OSEM reconstruction and a low-dose CT scan was done 60 min ± 10 after radiotracer injection. Screening for suspicious lesions on [^18^F]FDG PET/CT imaging was done by two nuclear medicine physicians (SB and NS) and patients with at least three suspicious lesions were considered eligible for [^99m^Tc]Tc-Tilmanocept imaging.

#### [^99m^Tc]Tc-Tilmanocept imaging

After physical examination by the referring oncologist, patients were addressed for [^99m^Tc]Tc-Tilmanocept SPECT/CT imaging authorized under the name Lymphoseek® for lymph node mapping (Swissmedic #66,824). Lymphoseek® was prepared in dedicated station to maintain aseptic conditions and radiation safety, it was supplied as kit with [^99m^Tc] radiolabeling and proper dilution with sterile 0.9% sodium chloride solution and quality control was performed prior to injection. After measurement of vital signs, patients received an intravenous bolus injection of 250 µg Tilmanocept labelled with 370 MBq of [^99m^Tc]. Patients had 30-min planar dynamic acquisition centred on the site of a tumour lesion identified by [^18^F]FDG PET/CT (anterior and posterior views: 128 × 128 matrix, zoom: 1.0, 120 images of 15 secs), immediately followed by a whole-body planar acquisition (anterior and posterior views) at 12 cm/s in auto-contour mode. Quantitative SPECT/CT acquisition was done at 1- and 3-h after [^99m^Tc]Tc-Tilmanocept injection (xSPECT/CT, Siemens Symbia Intevo, Erlangen, Germany), centred on the region of interest. The SPECT/CT acquisition parameters were as follows: 20 seconds × 60 images for one anatomical area (i.e. thorax alone) and 15 seconds × 60 images if at least two anatomical areas (i.e. thorax and abdomen) were imaged; low energy collimator LEHR: 140 keV energy peak with a 20% window and 256 × 256 matrix with a low-dose CT scan (130 kV; 40 mA).

### Imaging analysis

For each patient, we assessed the same three suspicious lesions defined as target lesion (T-Lesion) on [^18^F]FDG PET/CT and on [^99m^Tc]Tc-Tilmanocept SPECT/CT imaging at both 1- and 3-h. We measured in a spheric VOI after semi-automatic segmentation based on a 40% threshold of the maximum standard uptake value (SUV_max_): SUV_mean/max_, metabolic tumour volume (MTV) on both imaging modalities, whereas tumour lesion glycolysis (TLG) was measured on [^18^F]FDG PET/CT and tumour lesion activity (TLA) on [^99m^Tc]Tc-Tilmanocept SPECT/CT. The expected signal measured on tumour lesions on [^99m^Tc]Tc-Tilmanocept xSPECT/CT was expected to be low. As such, we aimed to increase its absolute value by using ratios between T-Lesion SUV_max_ and SUV_mean_ of non-tumoural tissues measured within a spherical volume of interest on SPECT/CT imaging at 1- and 3-h to determine the highest ratio between T-Lesion SUV_max_ and healthy tissue SUV_mean_. The non-tumoural tissues were as follows: satellite tissue just outside of the suspicious lesion, contralateral tissue (same tissue opposite to the lesion site), liver, spleen, blood pool (in the descending thoracic aorta lumen), bone (vertebral body of L3), muscle (gluteal muscles when possible) and the fat-tissue (subcutaneous fat near the gluteal muscles when possible).

### Biopsy and multiplex immunofluorescence staining

The methodology described hereafter for immunofluorescence staining and quantification of cell densities was previously validated in our institution and already published (19). In short, multiplexed staining was performed on 4-µm formalin-fixed paraffin-embedded tissue sections on automated Ventana Discovery Ultra staining module (Ventana, Roche). Slides were placed on the staining module for deparaffinization, epitope retrieval (64 min at 98 °C) and endogenous peroxidase quenching (Discovery Inhibitor, 8 min, Ventana). Multiplex staining consists in multiple rounds of staining. Each round includes non-specific sites blocking (Discovery Goat IgG and Discovery Inhibitor, Ventana), primary antibody incubation, secondary HRP-labelled antibody incubation for 16 min (Discovery OmniMap anti-rabbit HRP (Ventana, # 760–4311) or anti-mouse HRP (Ventana, #760–4310)), OPAL™ reactive fluorophore detection (Akoya Biosciences, Marlborough, MS, USA) that covalently label the primary epitope (incubation: 12 min) and then antibodies heat denaturation. Panel was optimized to look at the macrophages and CD8 T cells. Sequence of antibodies used in the multiplex with the associated OPAL are the following: 1st: mouse monoclonal anti-human PD1 antibody (2 µg/mL, NAT105, Biocare, 1-h, RT, dilution 1/1500), OPAL520; 2^d^: mouse anti-CD163 antibody (15.8 µg/mL, 10D6, Diagnostic Biosystem, 1-h, 37 °C, dilution 1/500), OPAL620; 3rd: rabbit anti-human CD206 antibody (0.1 mg/ml, HPA004114, Sigma, 1-h, 37 °C, dilution 1/100), OPAL570; 4th: mouse anti-CD68 antibody (40 mg/ml, Clone PG-M1, DAKO, 1-h, 37 °C, dilution 1/1000), OPAL480; 5th: rabbit anti-SOX10 antibody (245.5 µg/ml, EP268, Cellmarque, 1-h, 37 °C, dilution 1/200), OPAL690; 6th: rabbit anti-CD8 antibody (0.3 µg/mL, clone SP16, Cellmarque, 1-h, 37 °C, dilution 1/400), OPAL780. Nuclei were visualized by a final incubation with Spectral DAPI (1/10, FP1490, Akoya Biosciences) for 12 min. Multiplex immunofluorescence images were acquired on PhenoImager allowing a whole slide multispectral imaging acquisition (Akoya Biosciences). IF signal extractions from qptiff images were performed using our in-house developed software IFQuant (https://github.com/BICC-UNIL-EPFL/IFQuant), enabling a per-cell analysis of IF markers of multiplex stained tissue sections and counting of every cell’s population. Based on tumour cell marker (SOX10), the software automatically defined tumour area containing at least ten SOX10 positive cells, and stromal area containing at least ten SOX10 negative cells. To assess the tissue heterogeneity for several markers, we segmented the entire multispectral image into tiles. The size of each tile was set by an area-based criterion, typically 640,000 square microns. A shift of 2 (the tiles partially overlap at their centres) was applied to increase the granularity of tile positioning. By iteratively shifting the tile, we calculated the tissue area and phenotype cell count per tile. Median densities of tile are then computed for each sample in each tissue category (tumour and stroma). To determine the inflammation status based on CD8^+^ cells, a threshold density of 21 CD8^+^ cells per mm^2^ was set to determine infiltrated tumours with a higher presence of immune cells, hence labelled “inflamed” (> 21 CD8^+^ cells per mm^2^ in tumour) versus “excluded” if otherwise (> 21 CD8^+^ cells per mm^2^ in stroma and < 21 CD8^+^ cells per mm^2^ in tumour).

### Clinical data and follow-up

For each patient, we collected information on gender, age at the time of [^99m^Tc]Tc-Tilmanocept imaging, clinical history, initial location of the melanoma, TNM and Breslow staging, ICI treatment and clinical setting (baseline without prior ICI treatment or PD under ICI). Tumour response at three months was assessed on [^18^F]FDG PET/CT according to modified EORTC criteria (20, 21) with complete response (CR) defined as the regression of all suspicious metabolic lesions, partial response (PR) defined as a global diminution in SUV_max_ of at least 25% of TL without new lesions, whereas a global progression of all suspicious lesions of 25% of SUV_max_ and/or new lesions was considered as PD. If [^18^F]FDG PET/CT imaging at three months was not available, tumour response was assessed on conventional morphological imaging using RECIST 1.1 criteria (22)*.*

### Statistical analysis

We, respectively, assessed and compared the association between SUV_mean/max_, MTV, TLG/TLA measured in T-Lesion on [^99m^Tc]Tc-Tilmanocept SPECT at 1- and 3-h using Spearman correlation and t test for dependent samples.

Per lesion analysis: We used Spearman correlation to assess the association between SUV_mean/max_, MTV and TLA values of T-Lesion on [^99m^Tc]Tc-Tilmanocept SPECT/CT at 1- and 3-h and SUV_mean/max_, MTV and TLG values of T-Lesion on baseline [^18^F]FDG PET/CT. We looked at the correlation between SPECT/CT parameters and ΔSUV_mean/max_, ΔMTV and ΔTLG on the second [^18^F]FDG PET/CT at three months. Similar analyses were done using the highest ratio between T-Lesion SUV_max_ and SUV_mean_ of non-tumoural tissue on [^99m^Tc]Tc-Tilmanocept SPECT at 1- and 3-h. We compared total cell densities in cells/mm^2^ in the stroma and the tumour for CD206 + , CD206 + PD1 + , CD163 + 206 + , CD8 + and macrophages on biopsies of TL seen on [^99m^Tc]Tc-Tilmanocept SPECT/CT (or closest lesion to the biopsy site) to T-Lesion’s SUV_mean/max_, MTV, TLA and T-Lesion SUV_max_/SUV_mean_ fat-tissue ratio at 1-h. We used Mann–Whitney test to compare previously described cell densities according to tumour response at three months, the immune biopsy status and the outcomes during the follow-up.

Per patient analysis: We used Mann–Whitney test to compare SUV_max/mean_, MTV and TLA measured on T-Lesion on SPECT at 1- and 3-h according to the tumour response at three months on the second [^18^F]FDG PET/CT or contrast-enhanced CT (CE-CT) between “responders” (CR and PR) and “non-responders” (PD). Similar analysis were done according the immune biopsy status (“inflamed” versus “excluded”) and the outcomes during the follow-up (disease progression or recurrence versus no event).

Statistical analyses were done using IBM SPSS statistics 27.0.1.0 (*IBM*® *SPSS*® *Statistics)* with p value below 0.05 considered statistically significant.

### Ethics

This study was done according to the ethical standards defined by the Helsinki declaration and its later amendments. Each patient signed a consent form after receiving clear, complete and comprehensive information about the protocol which was approved by our local ethics committee (CER-VD: 2019–01763) and registered with clinicaltrials.gov (NCT04663126).

## Results

### Population

**Fig. 1:**
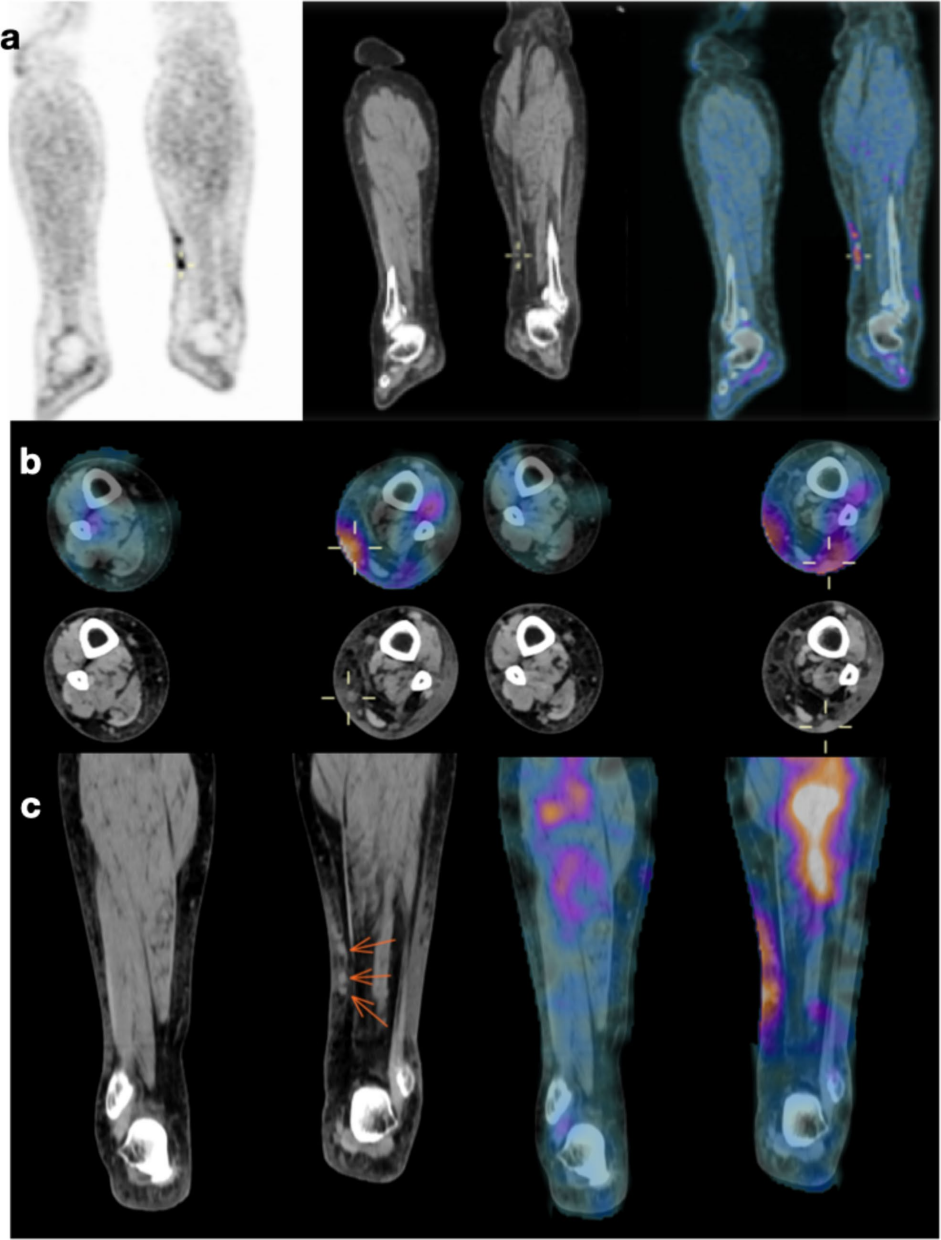
73-year woman with stage IIIC melanoma of the left calf melanoma and subcutaneous lesions: a) coronal views of PET image, CT image and fused [^18^F]FDG PET/CT image b) axial slices showing on top row fused [^99m^Tc]Tc-Tilmanocept SPECT/CT images at 1-h and CT images on bottom row c) coronal slices showing CT images with arrows pointing to subcutaneous lesions and fused [^99m^Tc]Tc-Tilmanocept SPECT/CT images at 1-h

We recruited five patients from May 2021 to January 2023 with a mean age of 75.9 ± 2.9 years (Table 1). Most patients (60%) were at initial staging at the time of the first [^18^F]FDG PET/CT scan and never received ICI treatment (Table 2). One patient (Patient 5) had a concomitant diagnosis of follicular lymphoma grade 1–2, BCL2 + that did not require specific treatment. All patients had [^99m^Tc]Tc-Tilmanocept SPECT/CT scans (at 1- and 3-h) within the month after the [^18^F]FDG PET/CT scan (21.8 ± 8.3 [8–29] days) that showed at least three suspicious lesions. Three identical T-Lesions were analysed on both imaging with a total of 15 T-Lesions: seven soft tissues lesions (Fig. 1, Supplemental Fig. 1, Table 3), three lymph nodes, three bone lesions and one pancreatic lesion.

**Table 1:** Patients’ characteristics; * N.A Not applicable, ** NGS—panel 400 genes

Patients	Age	Gender	Histology	Breslow	TNM/Stage
**Patient 1**	**73.1**	**Female**	**Melanoma left calf**	**Breslow 8 mm**	**pT4a cN1c cM0/IIIC**
*Patient 2*	*76.7*	*Male*	*Melanoma left pectoral*	*Breslow 14 mm*	*pT4b cN1 cM1/IV*
**Patient 3**	**80.1**	**Female**	**Melanoma unknown primary**	N.A *	**cTx cNx M1/ IV**
*Patient 4*	*73.1*	*Male*	*Melanoma lower back*	*Breslow 5.5 mm (ulcerated)*	*pT4b pN1a cM0/IIIC*
**Patient 5**	**76.7**	**Male**	**2 Melanomas (left infraorbital and presternal)**	**Breslow 1.2 mm and Breslow 10 mm**	**pT2a et pT4b pN2 (2/4) M1/IV**

**Table 2:** Treatment and follow-up

Patient	Baseline/under immunotherapy	Immunotherapy	Previous immunotherapy	Tumour response at three months	Follow-up
**Patient 1**	**Baseline**	**First-line pembrolizumab 200 mg/3w**	**No**	**Complete response**	**No disease recurrence**
*Patient 2*	*Baseline*	*First-line* *pembrolizumab 200 mg/3w*	*No*	*Partial response*	*Loss of follow-up*
**Patient 3**	**Baseline**	**First-line ipilimumab 3 mg/kg and nivolumab 1 mg/kg**	**No**	**Partial response**	**Progressive disease and new treatment**
*Patient 4*	*Under immunotherapy (1 cycle)*	*Second-line ipilimumab 3 mg/kg and nivolumab 1 mg/kg*	*First-line pembrolizumab 200 mg/3w (stopped irAE)*	*Dissociated response*	*Progressive disease and new treatment*
**Patient 5**	**Under immunotherapy (2 cycles)**	**Second-line ipilimumab 3 mg/kg and nivolumab 1 mg/kg**	**First-line pembrolizumab 200 mg/3w (stopped progressive disease)**	**Dissociated response**	**Dissociated evolution (limited PD) with immunotherapy continuation**

**Table 3:** Values for SUV_max/mean/peak_, TLG/TLA and MTV values measured on the initial [^18^F]FDG PET/CT scan and [^99m^Tc]Tc-Tilmanocept SPECT/CT at 1- and 3-h post-injection

Patient number	Lesion site	Baseline [^18^F]FDG PET/CT scan			[^99m^Tc]Tc-Tilmanocept SPECT/CT at 1-h				[^99m^Tc]Tc-Tilmanocept SPECT/CT at 3-h			
		SUV mean	SUV max	MTV	SUV mean	SUV max	MTV	SUVmax T-Lesion/SUVmean fat-tissue	SUV mean	SUV max	MTV	SUVmax T-Lesion/SUVmean fat-tissue
Patient 1	**Subcutaneous lesion lower limb**	*2.2*	*4.1*	*2.4*	***0.4***	***0.5***	***2.8***	***4.1***	*0.3*	*0.3*	*2.6*	*2.1*
	**Subcutaneous lesion lower limb**	*2.2*	*4.0*	*2.1*	***0.3***	***0.4***	***1.7***	***3.7***	*0.3*	*0.4*	*7.3*	*2.8*
	**Subcutaneous lesion lower limb**	*1.5*	*2.6*	*2.4*	***0.3***	***0.4***	***1.5***	***3.3***	*0.3*	*0.3*	*7.4*	*2.6*
Patient 2	**Subcutaneous lesion pectoral**	*13.3*	*21.6*	*1.5*	***0.4***	***0.6***	***3.8***	***3.6***	*0.4*	*0.6*	*5.1*	*3.8*
	**Axillary lymph node**	*8.9*	*14.1*	*2.5*	***0.6***	***1.1***	***2.9***	***6.7***	*0.5*	*0.9*	*3.7*	*5.4*
	**Sternum bone lesion**	*4.0*	*6.2*	*0.8*	***0.9***	***1.2***	***0.8***	***7.1***	*2.0*	*2.6*	*1.2*	*15.1*
Patient 3	**Subcutaneous lesion breast**	*7.1*	*12.3*	*2.4*	***0.5***	***0.8***	***1.0***	***4.5***	*0.3*	*0.5*	*0.9*	*4.8*
	**Subcutaneous lesion thorax**	*8.5*	*14.8*	*0.8*	***0.3***	***0.5***	***2.3***	***2.9***	*0.3*	*0.5*	*2.3*	*4.5*
	**Pancreatic lesion**	*6.3*	*11.4*	*10.9*	***1.2***	***2.1***	***8.5***	***11.6***	*0.8*	*1.3*	*5.1*	*11.9*
Patient 4	**Muscle lesion upper limb**	*3.0*	*5.2*	*1.7*	***0.3***	***0.6***	***2.1***	***9.2***	*0.2*	*0.4*	*3.2*	*3.7*
	**Lung pleural lesion**	*13.7*	*21.5*	*2.7*	***0.6***	***0.9***	***3.4***	***15.7***	*0.5*	*0.9*	*4.8*	*8.8*
	**Inguinal lymph node**	*6.3*	*10.4*	*1.4*	***0.6***	***0.9***	***0.8***	***15.7***	*0.5*	*0.7*	*0.5*	*7.2*
Patient 5	**Iliac bone lesion**	*9.3*	*14.3*	*2.4*	***1.0***	***1.7***	***2.3***	***8.7***	*1.0*	*1.7*	*1.2*	*13.4*
	**Axillary lymph node**	*10.2*	*16.3*	*7.5*	***0.8***	***1.5***	***5.2***	***7.8***	*0.6*	*1.2*	*8.1*	*8.9*
	**Sternum bone lesion**	*8.7*	*15.6*	*59.0*	***1.0***	***2.0***	***19.7***	***10.3***	*0.9*	*1.6*	*44.9*	*12.5*

### Imaging analysis

Dynamic images were acquired in all patients centred on a single anatomical region where one or several lesions were previously seen on baseline [^18^F]FDG PET/CT images but showed a low signal-to-noise ratio in all patients without significant counts measurable in the lesions (Supplemental Fig. 2).

**Fig. 2:**
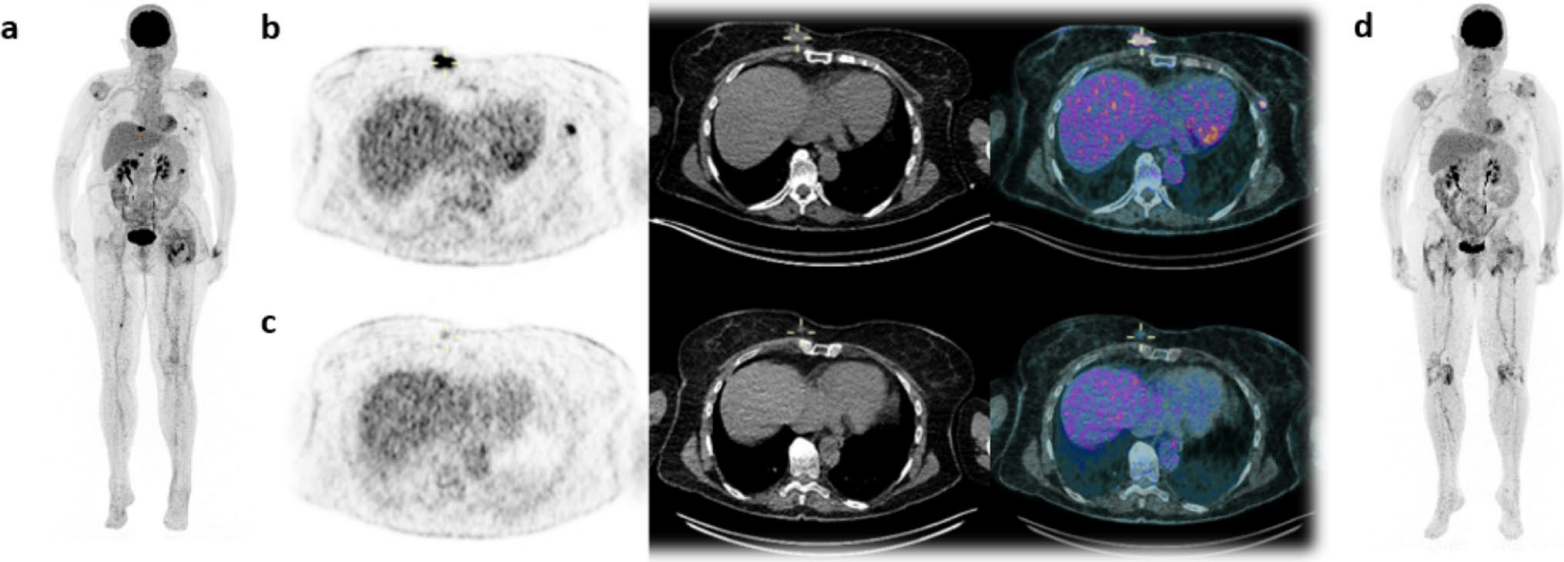
80-year woman with stage IV melanoma with unknown primary with multiple metastatic lesion: a and d) Maximum intensity projections [^18^F]FDG PET images for the first scan and second scan at three months, respectively; b) transaxial slices showing PET, low-dose CT and fused PET/CT images of the first [^18^F]FDG PET/CT scan, and c) transaxial slices showing PET, low-dose CT and fused PET/CT images of the second [^18^F]FDG PET/CT scan with a partial metabolic response

On whole-body planar imaging and on [^99m^Tc]Tc-Tilmanocept SPECT/CT imaging at both 1- and 3-h, physiological uptake can be seen predominantly in the liver, the spleen and the bone marrow with urinary excretion of the radiopharmaceutical (Supplemental Fig. 1).

There was no significant statistical difference for SUV_max/mean_, MTV and TLA measured in T-Lesions using t-test for dependent samples between 1-h and 3-h. Strong correlations between SUV_max/mean_ and MTV measured in T-Lesions on [^99m^Tc]Tc-Tilmanocept SPECT/CT at 1- and 3-h (Rho [0.71–0.93]; all p ≤ 0.003) but not for TLA (p = 0.32) without significant difference (all p > 0.15). SUV_max/mean_ values measured in T-Lesions on the initial [^18^F]FDG PET/CT scan were significantly higher than those measured on [^99m^Tc]Tc-Tilmanocept SPECT/CT at both 1- and 3-h with a tenfold ratio (all p < 0.001; Table 3). Similar results were found when comparing T-Lesions’ TLG and TLA (all p < 0.001). There were, however, significant correlations between SUV_max/mean_, MTV and TLG/TLA measured in T-Lesion on [^18^F]FDG PET/CT and [^99m^Tc]Tc-Tilmanocept SPECT/CT at 1- (Rho = [0.53–0.81]; all p ≤ 0.04) and 3-h (Rho [0.52–0.60]; all p ≤ 0.045). The highest ratio found between T-Lesion SUV_max_ and SUV_mean_ of non-tumoural tissues on SPECT/CT imaging was with fat-tissue at both 1- and 3-h and all the ratios remained stable between 1- and 3-h (Supplemental Table 1). On a per lesion analysis, there was no significant association between SUV_max/mean_, MTV and TLA measured on SPECT at 1- and 3-h and ΔSUV_max/mean_, ΔMTV and ΔTLG in the four patients that had a second [18F]FDG PET/CT.

### Correlations with Immunofluorescence biopsies

**Figures 3:**
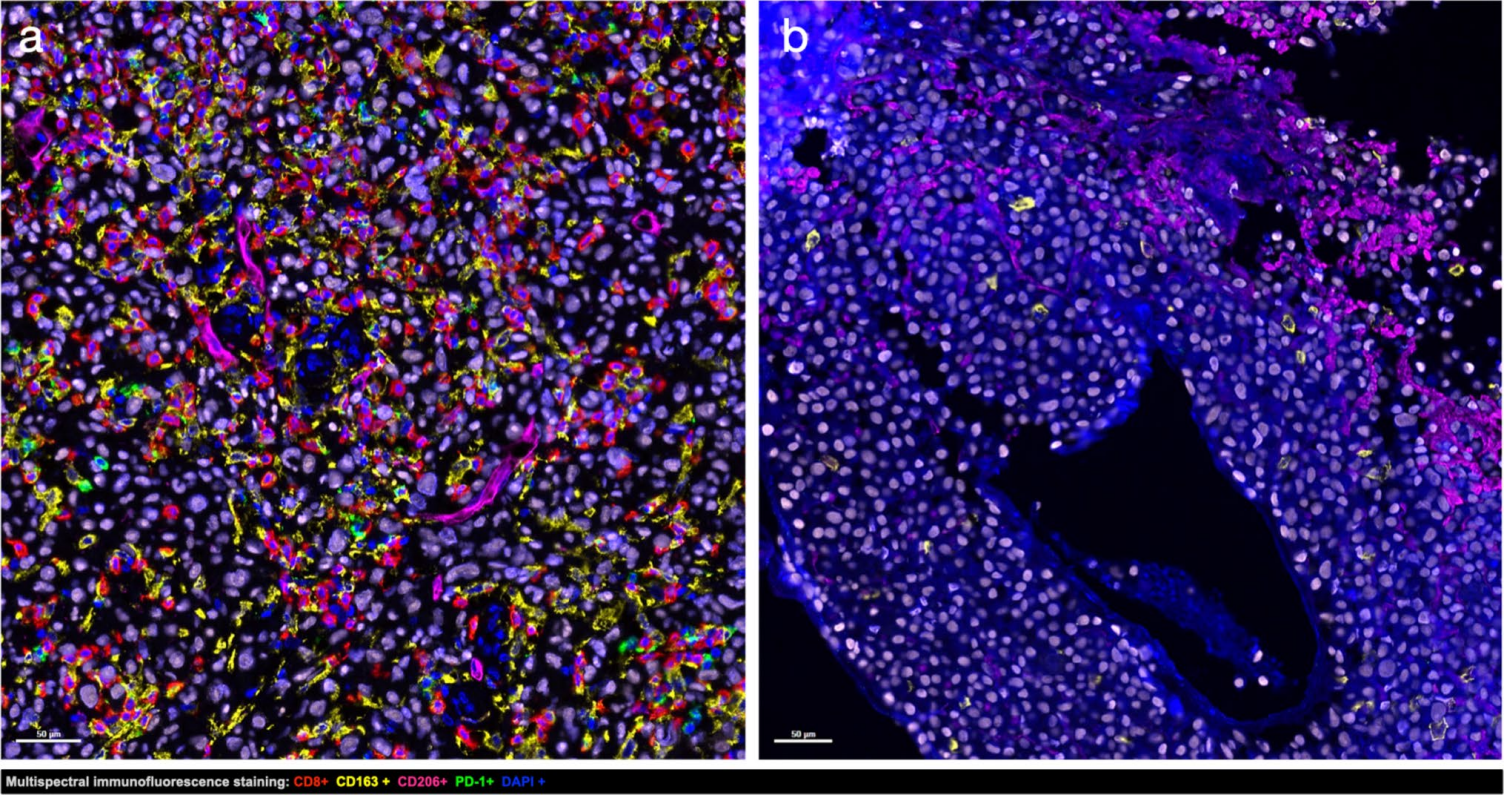
Multispectral immunofluorescence staining images in red CD8 staining, in green PD1 staining, in yellow CD163 staining, in pink CD206 staining, in light pink SOX10 staining, and in blue DAPI staining: a) 73-year woman with stage IIIC melanoma of the left calf melanoma with high immune cells infiltration “inflamed” and b) 77-year man with stage IV melanoma with two primary melanoma locations (left infraorbital and presternal) with low immune cells infiltration “excluded”

Biopsy data were available in all patients with a total of eight biopsies (patient 4 had three biopsies and patient 5 had two biopsies), out of which six biopsies were assessed as a function of imaging findings (Figs. 3a and 3b; Table 4). Significant associations between cells densities and T-Lesions’ uptake on SPECT/CT imaging were found between SUV_max_ T-Lesion/SUV_mean_ fat-tissue ratio and CD8 + Total in the stroma and macrophages in the tumour (Rho = -0.89 and 0.89, respectively; all p = 0.03). There was also a borderline association between SUV_max_ TL/ SUV_mean_ fat-tissue ratio and CD8 + Total in the tumour (Rho = -0.77; all p = 0.1) and MTV values measured on SPECT/CT at 1-h and CD206 + PD1 + total cells density in the stroma (Rho = 0.66; p = 0.18). There were no significant association between T-Lesion uptake on [^99m^Tc]Tc-Tilmanocept SPECT/CT at 1-h and CD206 + , CD206 + PD1 + or CD163 + 206 + cell densities in the stroma or the tumour. There were significant correlations between ΔSUVmax on the second [^18^F]FDG PET/CT at three months and total cells densities for CD8 + in the stroma and macrophages in the tumour (Rho = -1 and 1, respectively; all p = 0.017) and a borderline association between CD8 + total cells density in the tumour (Rho = -0.90; p = 0.08). There was also a borderline association between SUV_max_ T-Lesion/ SUV_mean_ fat-tissue ratio and the immune biopsy status with higher ratios in “excluded” biopsy status in comparison with “inflamed” (11.7 ± 3.5 versus 3.5 ± 0.6, respectively; p = 0.1).

**Table 4:** Values for cell density in cells/mm2 measured for CD206 + , CD206 + PD1 + , CD163 + 206 + , CD8 + and macrophages using multispectral immunofluorescence staining in the stroma and the tumour; immune biopsy status is also mentioned

Patient number	Lesion biopsy site	CD206 + Total		CD206 + PD1 + Total		CD163 + 206 + Total		CD8 + Total		Macrophages total		Immune biopsy “inflamed” versus “excluded”
		Stroma	Tumour	Stroma	Tumour	Stroma	Tumour	Stroma	Tumour	Stroma	Tumour	
Patient 1	Subcutaneous lesion lower limb	**165.56**	**16.68**	**1.9**	**0**	**1.13**	**0**	**728.26**	**104.08**	**2692.85**	**270.83**	Inflamed
Patient 2	Subcutaneous lesion pectoral	**191.1**	**1.9**	**16.1**	**0**	**9.7**	**0**	**1866.4**	**152.4**	**3604.4**	**75**	Inflamed
Patient 3	Subcutaneous lesion thorax	**309.28**	**11.11**	**6**	**0.04**	**2.19**	**0.03**	**1010.6**	**47.07**	**2714.53**	**180.02**	Inflamed
Patient 4	Subcutaneous lesion upper limb	**59.874**	**11.92**	**0.54**	**0**	**0.317**	**0**	**43.272**	**11.97**	**6919.859**	**622.71**	Excluded
	Inguinal lymph node	**253.335**	**7.13**	**4.559**	**0.019**	**3.006**	**0.012**	**26.351**	**3.361**	**6067.793**	**688.815**	Excluded
Patient 5	Sternal bone lesion	**2129,6**	**260.4**	**15.9**	**0.8**	**2.1**	**0**	**3.7**	**0**	**5452.5**	**688.9**	Excluded

### Tumour response assessment

All patients had an assessment of the tumour response at approximatively three months after the initial [^18^F]FDG PET/CT scan 3.5 ± 1.3 [2.1–5.5] months. Four patients had a second [^18^F]FDG PET/CT scan and one patient (Patient 3) had a CE-CT scan at 5.5 months. Most patients had a good tumour response, one patient (Patient 1) presented with CR and two patients had PR (Patients 2 and 3, Fig. 2). Conversely, two patients were classified as non-responders with PD (Patients 4 and 5) due to the appearance of new suspicious lesions despite T-Lesion meeting the criteria for PR on a lesion basis: mean decrease in ΔSUV_max_ of -75.3% ± 12.4 and 60.9% ± 17.8, respectively, suggesting a dissociated response. There was no significant difference for TL’s SUV_max/mean_, MTV and TLA on SPECT/CT at 1- and 3-h according to the global EORTC tumour response at three months (all p > 0.15). However, there were significantly higher ratios between SUV_max_ T-Lesion/SUV_mean_ fat-tissue at 1-h in non-responder patients 11.2 ± 1.3 versus 5.3 ± 2.8 (p = 0.005). On a per patient analysis, there were borderline associations between tumour response and total cells densities for CD8 + and macrophages in both the stroma and the tumour (all p = 0.1).

### Follow-up

**Figures 4:**
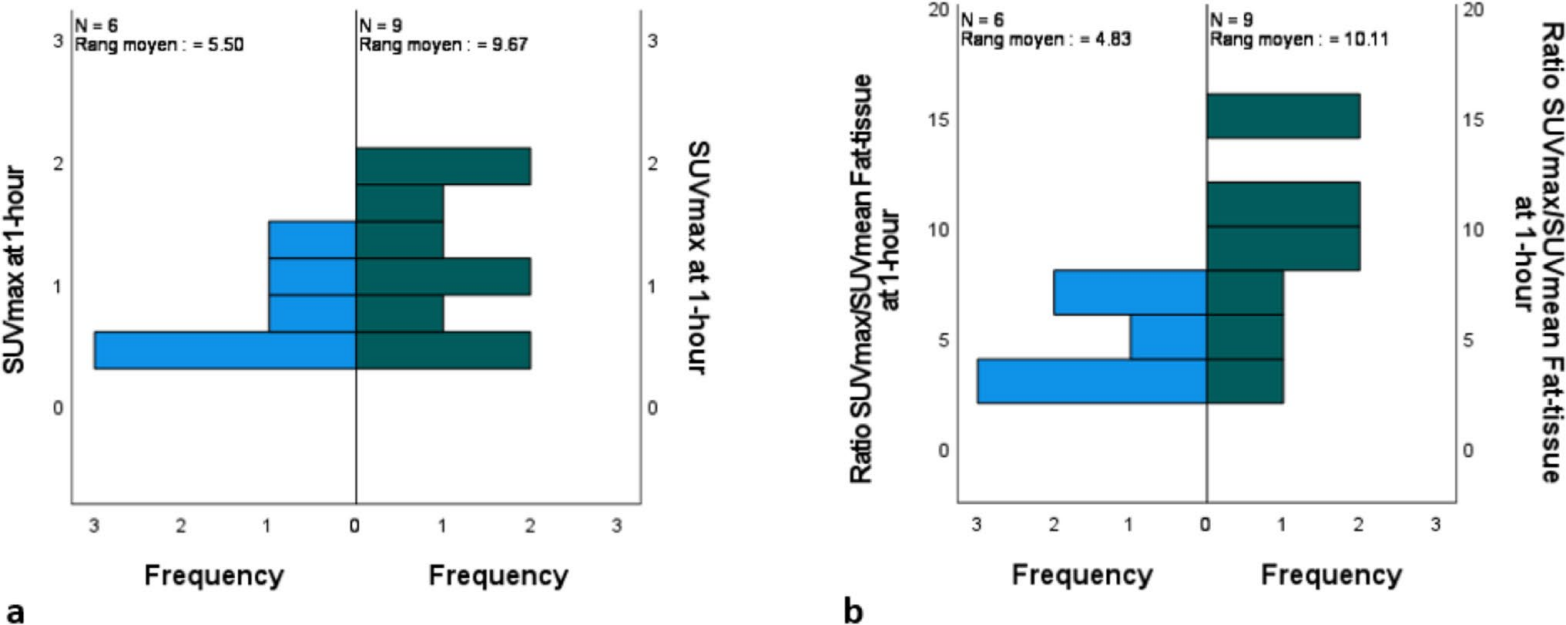
Graphics showing the differences in a) SUV_max_ values and b) ratio between SUV_max_ target lesion/ SUV_mean_ fat-tissue on [^99m^Tc]Tc-Tilmanocept SPECT/CT at 1-h between lesions in patients that presented with poor outcomes (in green) during the follow-up after the second [^18^F]FDG PET/CT scan and those that did not have disease recurrence or disease progression (in blue)

We had an average follow-up time of 14.4 ± 7.2 [6.8–25] months after the first [^18^F]FDG PET/CT scan. During the follow-up, three patients out of five presented with disease progression (Patients 3, 4 and 5). For two patients, it led to a change in treatment whereas for one patient (Patient 5) with limited disease progression and a persistent dissociated response, ICI treatment was maintained. When comparing SUV_max/mean_, MTV and TLA measured in T-Lesion on SPECT/CT imaging as a function of the outcomes, we did not find significant differences, though there was a tendency for a higher TL’s SUV_max_ on [^99m^Tc]Tc-Tilmanocept SPECT/CT at 1-h (p = 0.09; Fig. 4a). But, there were significantly higher ratios between SUV_max_ T-Lesion/ SUV_mean_ fat-tissue at 1-h in patients with poorer outcomes 9.6 ± 4.4 versus 4.7 ± 1.7 (p = 0.026; Fig. 4b). Looking at multispectral immunofluorescence staining findings, except for a borderline association between CD8 + total cells density in the tumour (p = 0.13) there was no significant association with outcomes.

## Discussion

This prospective feasibility study investigated the utility of CD206-based imaging of M2-like macrophages in patients with locally advanced or metastatic melanoma undergoing ICI treatment. We showed measurable imaging signal of secondary lesions seen on baseline [^18^F]FDG PET/CT in melanoma patients using [^99m^Tc]Tc-Tilmanocept SPECT/CT. First, looking at the imaging protocol using [^99m^Tc]Tc-Tilmanocept, we found that dynamic imaging reflecting initial perfusion had a very low signal-to-noise ratio and was not clinically relevant, hence could be removed from further studies. Initially, quantitative SPECT/CT imaging was planned at 1-h p.i. to evaluate hyperaemia, and delayed imaging at 3-h aimed to measure CD206-mediated tracer uptake. However, T-Lesion’s signal remained stable over time with strong correlations and without significant differences for SUV_max/mean_, MTV and TLA between time points and questioning the utility of maintaining the additional 3 h SPECT/CT acquisition. Additionally, it would be easier to translate in routine practice to limit patients' imaging time to a quantitative SPECT/CT imaging at 1-h after WB body planar imaging. All the more so as we showed significant correlations between T-Lesions’s uptake on [^99m^Tc]Tc-Tilmanocept SPECT/CT at 1- and 3-h and on baseline [^18^F]FDG PET/CT. However, [^18^F]FDG uptake is not specific to cancer cells as inflammatory cells such as macrophages also have a higher glucose metabolism. Thus, T-Lesion uptake on [^18^F]FDG PET/CT likely might reflects both tumour and inflammatory cells in the TME, whereas [^99m^Tc]Tc-Tilmanocept uptake is supposed to be more specific to macrophages (23).

The highest ratio between T-Lesion SUV_max_ and SUV_mean_ non-tumoural that was with fat-tissue leading to an increase in the absolute signal values for T-Lesion uptake on [^99m^Tc]Tc-Tilmanocept SPECT/CT. That ratio on [^99m^Tc]Tc-Tilmanocept SPECT/CT at 1-h was associated with tumour response at three months in this small group of patients with higher ratios in “non-responders” patients. However, we should mention that the two patients classified as non-responders with PD because the appearance of new suspicious lesions on the second [^18^F]FDG PET/CT had a significant decrease in T-Lesion [^18^F]FDG uptake suggesting a dissociated response. However, the clinical significance of a dissociated response is not consensual and most patients will likely present with poor outcomes during the follow-up as it happened in our study. New classifications suggested new criteria to assess dissociated response in patients treated with ICI without definite consensus and in our small cohort we chose not to investigate it (24–26). Similarly to poorer tumour response, highest ratios on SPECT/CT at 1-h were seen in the three patients that presented with poorer outcomes during the follow-up suggesting a potential clinical benefit [^99m^Tc]Tc-Tilmanocept imaging to predict outcomes in patients treated with ICI.

Looking at multiplex immunofluorescence staining findings, a significant positive correlation was found between macrophages cells densities of in the tumour and SUV_max_ T-Lesion SUV_max_/SUV_mean_ fat-tissue ratio measured on [^99m^Tc]Tc-Tilmanocept SPECT/CT at 1-h confirming again the clinical relevance of this time-point. There was, however, no significant association found between cells densities for CD206 + , CD206 + PD1 + or CD163 + 206 + in the stroma or in the tumour and T-Lesion uptake on [^99m^Tc]Tc-Tilmanocept SPECT/CT at 1-h. We hypothesized that it might be related to a lower sensitivity of [^99m^Tc]Tc-Tilmanocept SPECT/CT to detect cell populations with lower cell densities with over a tenfold ratio for cell densities between macrophages and CD206 + total cells. The small number of biopsies available for this correlation was also a limitation. Conversely, negative associations were found with total cells densities of CD8 + cells in the tumour and in the stroma. The inverse associations found for total macrophages and CD8 + cells were in agreement with the literature (27). Interestingly, we also found a borderline association between SUV_max_ T-Lesion/SUV_mean_ fat-tissue ratio and immune biopsy status. Indeed, higher ratios were found in “excluded” in comparison with “inflamed” lesions on biopsy, hence associated with a higher macrophages and lower CD8 + cells population for the former and inversely for the latter. This could be relevant in further studies as there was a trend for an association between CD8 + cells densities tumour response at three months as well as patients’ outcomes though it was not statistically significant in this study. This association has been reported in the literature and several imaging studies investigated the prediction of tumour response in patients undergoing ICI treatment. Bensch et al. investigated [^89^Zr]-Atezolizumab-based PD-L1 imaging and found that tumour uptake but not histological PD-L1 expression predicted patients’ survival under ICI treatment with an extremely inhomogeneous SUV_max_ distribution in the same patient (28). A different study investigated PD-L1 expression in cancer patients using a ^18^F-labelled peptide, and showed a higher tracer uptake was significantly associated with local tumour response (29). Although these studies hold high potential for clinical implementation in the future, we presented here a new imaging concept to visualize immunosuppressive component of the TME, macrophages such as M2-like macrophages which presence increases the local infiltration with regulatory T cells (30, 31), another antagonistic component against ICI in the TME. Thus, [^99m^Tc]Tc-Tilmanocept imaging might be an ideal marker for measuring the presence of antagonistic cells in T-Lesion TME. Gondry et al. showed the feasibility of CD206-targeted PET imaging of macrophages using [^68^ Ga]Ga-NOTA-anti-CD206-sdAb in seven patients with solid tumours, confirming the good tolerance and safety of CD206-targeted tracers but did not investigate the outcomes and contrary to [^99m^Tc]Tc-Tilmanocept that is commercially available [^68^ Ga] labelling might be less common (32). Nonetheless, macrophages specific tracers might be useful in routine practice before initiating ICI. Indeed, it might help predict which eligible patient might derive the greatest clinical benefit from anti-PD1 therapy and further studies with a lager court could define the threshold for SUV_max_ T-Lesion/SUV_mean_ fat-tissue ratio on [^99m^Tc]Tc-Tilmanocept SPECT/CT at 1-h associated with poorer tumour response at three months or outcomes during the follow-up. This would be especially valuable as contrary to biopsies, [^99m^Tc]Tc-Tilmanocept imaging could provide with a wide and non-invasive in vivo assessment of patients’ secondary lesions potentially providing with information on their immune status related to CD206 + cells, such as M2-like macrophages cell densities known in the literature as predictor of adverse events (12).

We should nonetheless acknowledge main limitations in this study which that were the small sample size and the absence of a control group. We aimed to recruit 20 patients, but the COVID-19 pandemic hindered recruitment and we had to amend the inclusion criteria to include patients with PD under ICI, as the number of ICI-naïve patients was limited leading to a slightly heterogeneous population which undermined the statistical power of our results and limits their generalisability. Despite these limitations, in our opinion this study provided valuable proof-of-concept results, warranting further investigation in a bigger cohort of patients into the usefulness of CD206-based imaging in selecting patients scheduled for ICI treatment.

## Conclusion

This prospective feasibility study, first described the potential of CD206-based imaging in melanoma patients referred for immunotherapy as a proof of concept for potential clinical application. We showed measurable imaging signal of secondary lesions seen on baseline [^18^F]FDG PET/CT in melanoma patients using [^99m^Tc]Tc-Tilmanocept SPECT/CT with positive correlation between T-Lesion signal [^99m^Tc]Tc-Tilmanocept uptake and the clinically indicated baseline [^18^F]FDG PET/CT. Interestingly, [^99m^Tc]Tc-Tilmanocept imaging showed a highest signal in T-Lesions with a highest macrophages cells densities in the tumour on biopsy, thus potentially reflecting T-Lesion immune status. Our results also suggest a likely association between [^99m^Tc]Tc-Tilmanocept uptake on melanoma lesions and patients’ outcomes for tumour response at three months and during the follow-up. Nonetheless, those results are preliminary and its potential usefulness for selecting patients’ scheduled for immunotherapy should be further investigated in larger cohorts of patients.

## Supplementary Information

Below is the link to the electronic supplementary material.

Supplementary file1 (JPEG 468 KB)

Supplementary file2 (JPEG 196 KB)

Supplementary file3 (DOCX 10 KB)

## Data Availability

No datasets were generated or analysed during the current study.

## Abbreviations

CE-CT: Contrast-enhanced CT
CR: Complete response
ICI: Immune checkpoint inhibitor
MTV: Metabolic tumour volume
PD: Progressive disease
PD1: Programmed death-1 receptor
PD-L1: PD1 ligand
PR: Partial response
SPECT/CT: Single-photon emission tomography/computed tomography
SUV: Standard uptake value
TAMs: Tumour-associated macrophages
TLA: Tumour lesion activity
T-Lesion: Target lesion
TLG: Tumour lesion glycolysis
TME: Tumour micro-environment
[^18^F]-FDG PET/CT: [^18^F]-fluorodeoxyglucose Positron emission tomography/computed tomography
