# Supplementary figures and images for: Utility of [99mTc]Tc-tilmanocept, an immunosuppressive macrophage functional imaging agent in melanoma patients receiving checkpoint inhibitor treatment: a feasibility study
Source: Cancer Immunol Immunother. 2025 Sep 6;74(10):298. doi: 10.1007/s00262-025-04127-8 (PMC12414091; doi:10.1007/s00262-025-04127-8)

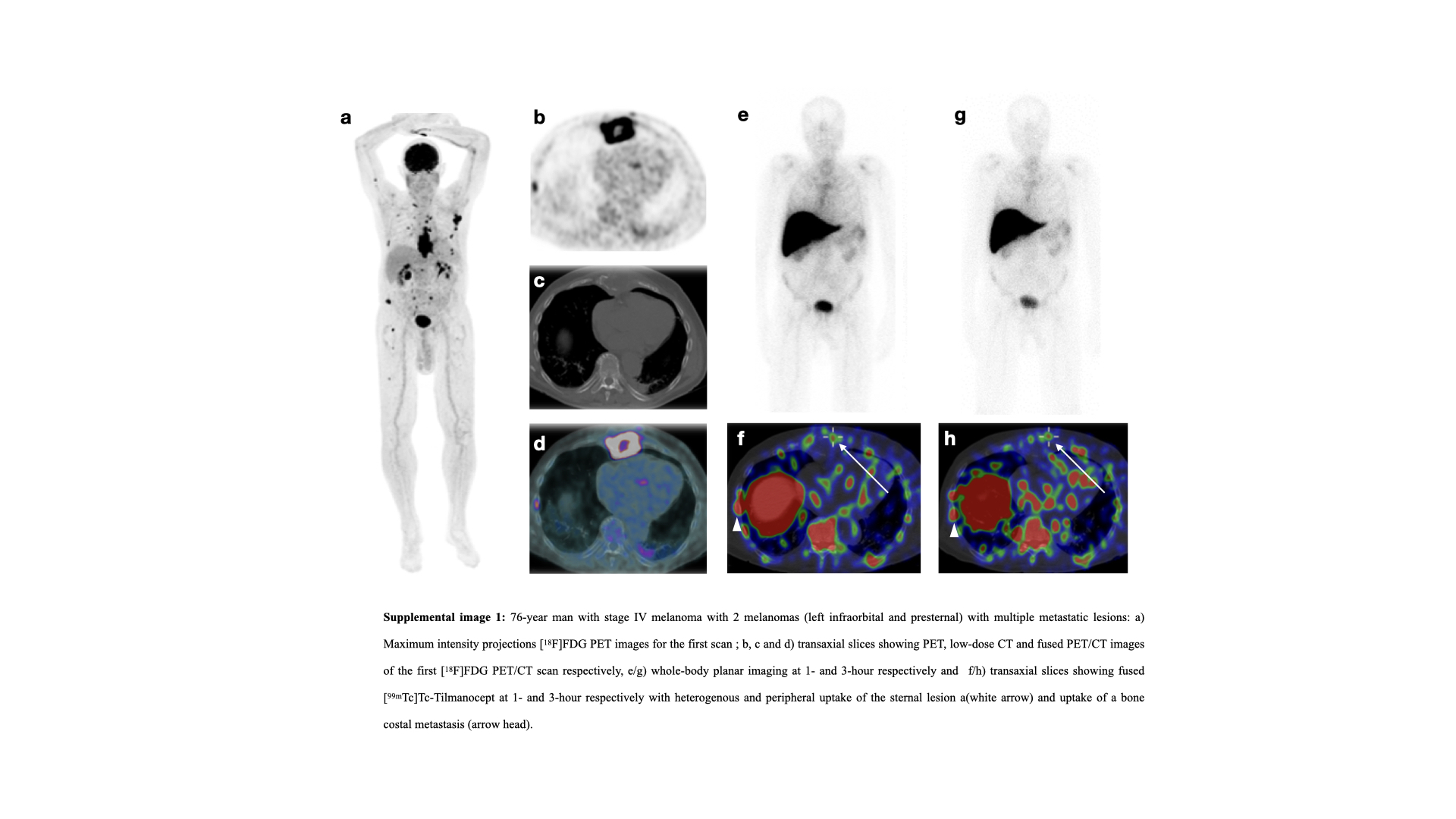

Supplement: Supplementary file 1 — Supplementary file1 (JPEG 468 KB) [file 262_2025_4127_MOESM1_ESM.jpeg]

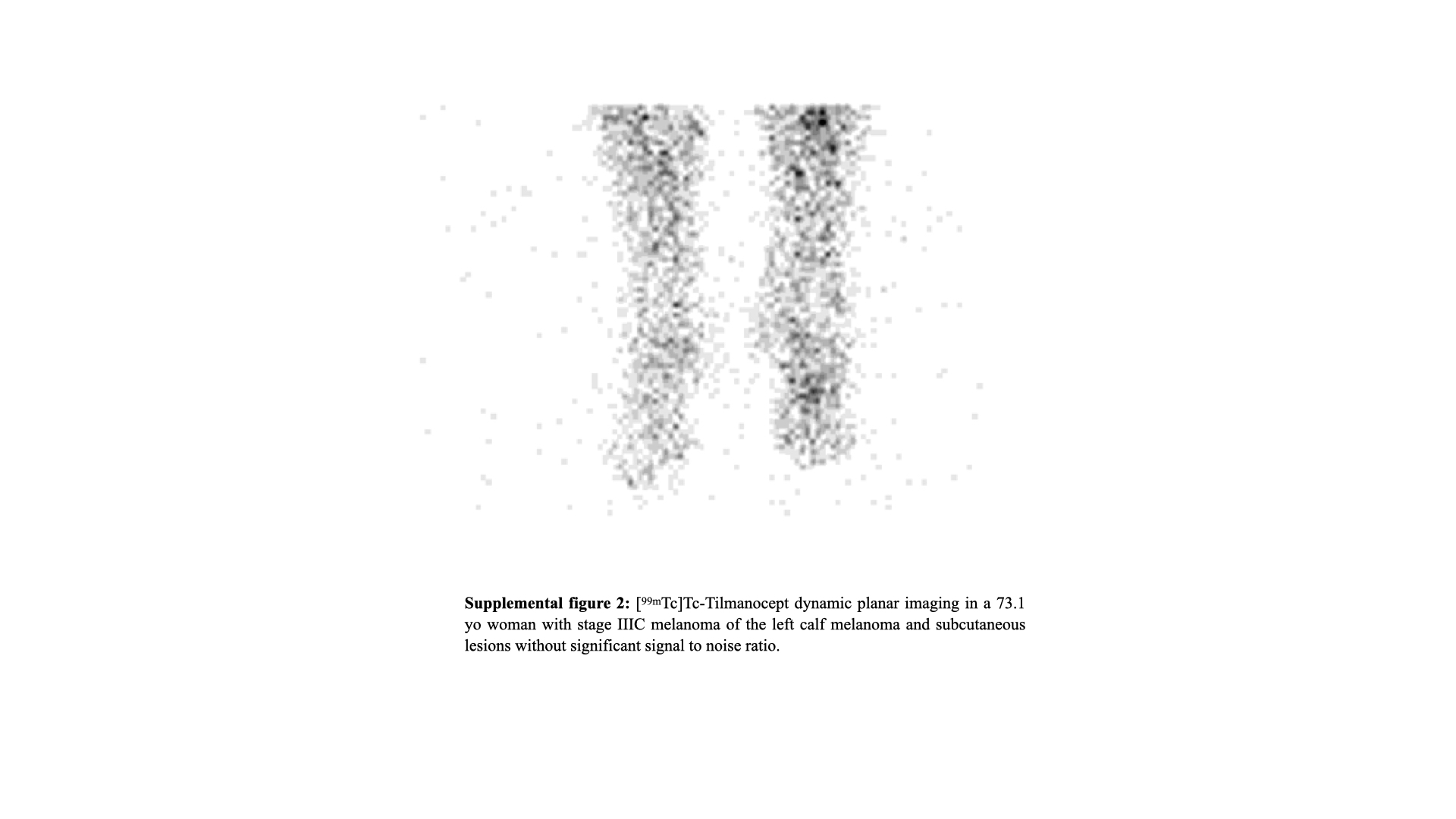

Supplement: Supplementary file 2 — Supplementary file2 (JPEG 196 KB) [file 262_2025_4127_MOESM2_ESM.jpeg]
