# Supplementary material for: Utility of [99mTc]Tc-tilmanocept, an immunosuppressive macrophage functional imaging agent in melanoma patients receiving checkpoint inhibitor treatment: a feasibility study
Source: Cancer Immunol Immunother. 2025 Sep 6;74(10):298. doi: 10.1007/s00262-025-04127-8 (PMC12414091; doi:10.1007/s00262-025-04127-8)
Supplement: Supplementary file 3 — Supplementary file3 (DOCX 10 KB) [file 262_2025_4127_MOESM3_ESM.docx]

| **Ratios T-Lesion SUV_max_/SUV_mean_ Healthy tissues** | | **Satelite-tissue** | **Controlateral-tissue** | **Muscle-tissue** | **Bone-tissue** | **Fat-tissue** | **Liver** | **Spleen** | **Blood pool** |
| --- | --- | --- | --- | --- | --- | --- | --- | --- | --- |
| At 1-hour | Mean±SD | 2.2±1.2 | 3.2±2 | 2.9 ±2.6 | 0.9 ±1.2 | **7.7±4.2** | 0.05 ±0.2 | 0.2±0.1 | 1.0±0.5 |
|  | Min | 0.8 | 1.0 | 0.1 | 0.1 | **2.9** | 0.02 | 0.1 | 0.4 |
|  | Max | 4.5 | 7.4 | 7.0 | 3.5 | **15.7** | 0.09 | 0.3 | 1.8 |
| At 3-hour | Mean±SD | 2.5±1.7 | 3.4±3.4 | 2.5 ±2.6 | 0.8 ±0.8 | **7.2±4.3** | 0.04 ±0.02 | 0.1±0.1 | 1.1±0.8 |
|  | Min | 0.7 | 0.9 | 0.1 | 0.1 | **2.1** | 0.02 | 0.1 | 0.3 |
|  | Max | 5.9 | 14.5 | 8.3 | 2.6 | **15.1** | 0.10 | 0.3 | 2.7 |

**Supplemental Table 1:** Mean values for ratios SUV_max_ T-Lesion over SUV_mean_ Healthy tissues measured on [^99m^Tc]Tc-Tilmanocept SPECT/CT at 1- and 3-hour post-injection ; highest ratio in bold.
